# A peptide encoded by a highly conserved gene belonging to the genus *Streptomyces* shows antimicrobial activity against plant pathogens

**DOI:** 10.3389/fpls.2023.1250906

**Published:** 2023-10-05

**Authors:** Byeong Jun Jeon, Nayeon Yoo, Jeong Do Kim, Jaeyoung Choi

**Affiliations:** ^1^ Smart Farm Research Center, Korea Institute of Science and Technology, Gangneung, Republic of Korea; ^2^ Department of Plant Biotechnology, Korea University, Seoul, Republic of Korea; ^3^ Department of Oriental Medicine Biotechnology, College of Life Sciences, Kyung Hee University, Yongin, Republic of Korea

**Keywords:** antimicrobial peptide, *Streptomyces*, plant pathogen, highly conserved sequence, antimicrobial activity

## Abstract

The genus *Streptomyces* has been unceasingly highlighted for the versatility and diversity of the antimicrobial agents they produce. Moreover, it is a heavily sequenced taxon in the phylum Actinobacteria. In this study, 47 sequence profiles were identified as proteins highly conserved within the genus *Streptomyces.* Significant hits to the 38 profiles were found in more than 2000 *Streptomyces* genomes, 11 of which were further conserved in more than 90% of Actinobacterial genomes analyzed. Only a few genes corresponding to these sequence profiles were functionally characterized, which play regulatory roles in the morphology and biosynthesis of antibiotics. Here a highly conserved sequence, namely, SHC-AMP (*Streptomyces* highly conserved antimicrobial peptide), which exhibited antimicrobial activity against bacterial and fungal plant pathogens, was reported. In particular, *Arabidopsis thaliana* was effectively protected against infection with *Pseudomonas syringae* pv. *tomato* DC3000 by treatment with this peptide. Results indicated the potential application of this peptide as an antimicrobial agent for control of plant diseases. Our results suggest putative target genes for controlling *Streptomyces* spp., including the one exhibiting antimicrobial activity against a wide range of phytopathogens.

## 1 Introduction

Natural products isolated from microorganisms have been regarded as an attractive source for the discovery of new pesticides (Patridge et al., 2016). The chemical and biological properties of natural products meet the recent requirements for environmentally friendly pesticides (Berdy, 2005). Natural products provide a wide spectrum of pharmacophores and a variety of stereochemistry because of their inherent scaffold diversity and unique functional groups as compared with synthetic compounds (Harvey et al., 2015). They likely lack cross-resistance to commercial pesticides. In addition, they may not cause residual problems of synthetic agents as natural products are biodegradable (Porter, 1985). Thus, natural products have been screened to discover new lead compounds with new mode of actions and biodegradability in natural environments.

Recently, antimicrobial peptides (AMPs) isolated from a wide range of taxonomy have shown great application potential in determining a new class of antimicrobial drugs because of their enormous unknown chemical diversity (O’Keefe, 2001). They possess a large and diverse subset of pharmacological space because of their structural diversity and complexity (O’Keefe, 2001). In addition, the broad-spectrum antimicrobial activities of AMPs suggest their potential benefits for treating various plant diseases. For example, thionins exhibit antimicrobial activity against plant pathogenic bacteria, fungi, and yeast (Bohlmann, 1999). The overexpression of plant thionin genes increased their resistance to bacterial wilt, Fusarium wilt, and black rot disease (Chan et al., 2005; Muramoto et al., 2012). Recombinant cecropin (*Musca domestica* mature cecropin) was reported to exhibit antifungal activity at the micromolar level against plant pathogenic fungi such as *Botrytis cinerea*, *Colletotrichum orbiculare*, and *Fusarium oxysporum* (Xu et al., 2007). Cationic peptides such as cecropin play an important role in antimicrobial activity, and they are widespread in nature. Their microbicidal action occurs via electrostatic interactions and/or membrane perturbation with the target cell. Positively charged peptides can interact strongly with negatively charged phospholipids within bacterial membrane structures, such as phosphatidylglycerol (PG), cardiolipin, lipopolysaccharide (LPS), and teichoic acid (Scott et al., 1999). Therefore, cationic AMPs readily form complexes with negatively charged phospholipids and induce a rapid killing effect after initial contact with the target cell membrane.

With the recent advances in DNA sequencing technology, publicly available genomes have increased exponentially and revealed a multitude of previously unknown microorganisms, potentially harboring pharmaceutically active molecules (Khabthani et al., 2021). Moreover, the accessibility of a “bottom-up approach” can facilitate investigations of genes, proteins, and pathways in a systems context (Park et al., 2021). *Streptomyces* spp. bacteria have been extensively studied in genome analysis during the last two decades because of their production of numerous extracellular enzymes and metabolic products (Gao and Gupta, 2005; van Bergeijk et al., 2020), providing a valuable resource for the discovery of novel AMPs. AMPs are evolutionarily conserved molecules involved in protective and therapeutic mechanisms against pathogen infection or invasion, and they can be used to identify novel molecular properties and taxonomic markers that are distinctive of *Streptomyces* spp.

The culture filtrate from *Streptomyces xanthophaeus* strain KPP03845 was found to effectively harbor an AMP against plant pathogens. In addition, a total of 19 AMP candidates that are highly conserved among 2062 *Streptomyces* genomes, including the strain KPP03845, were identified. A highly conserved sequence, namely, SHC-AMP (*Streptomyces* highly conserved antimicrobial peptide), exhibited antibacterial and antifungal activities against various plant pathogens and effectively protected *Arabidopsis thaliana* against *Pseudomonas syringae* pv. *tomato* DC3000 infection.

## 2 Materials and methods

### 2.1 Identification of pipeline for scanning potential AMPs from highly conserved proteins in the genus *Streptomyces*

A total of 1720 proteins were retrieved from NCBI Identical Protein Groups (IPG) by applying the following filters: Search *Streptomyces* Filters: RefSeq; Prokaryotes; >100. Only the sequences showing hits in ≥ 100 *Streptomyces* genomes were retained for further analysis. Ribosomal proteins and sequences of ≥100 aa were discarded. Protein sequences for the resulting 125 IPGs were clustered using mcl (v14-137) (Li et al., 2003) to reduce the dimension of search space. The analysis yielded 44 clusters, three of which were further separated into two subclusters based on manual inspection, yielding 47 total clusters (**Supplementary Table 1**). The prediction of potential AMPs was performed using Antimicrobial Peptide Scanner (v2), a prediction tool implemented in the database of antimicrobial activity and peptide structure (DBAASP v3.0), and AMPDiscover (Veltri et al., 2018; Pinacho-Castellanos et al., 2021; Pirtskhalava et al., 2021). Nucleotide sequences belonging to each subcluster were aligned by MUSCLE (v5.1) (Edgar, 2021) using default parameters. Hidden Markov model (HMM) sequence profiles were built and used to search 2061 *Streptomyces* genomes and 761 selected Actinobacterial genomes (**Supplementary Tables 2, 3**) (Eddy, 2011; Wheeler and Eddy, 2013). In visualizing the results of homology searches, a phylogenomic tree based on whole proteome sequences was created by using CVTree (Qi et al., 2004). The *K*-tuple length was set to six, as determined in the previous study (Zuo et al., 2010). The distribution of protein homology was visualized by a phylogenomic tree using Graphical Phylogenetic Analysis (GraPhlAn v1.1.4) (Asnicar et al., 2015).

### 2.2 Genome assembly, gene prediction, and functional annotation

The PacBio reads were assembled by RS HGAP Assembly (v3.0; Pacific Biosciences; https://www.pacb.com/products-and-services/analytical-software/smrt-analysis/). Pilon (v1.21) (Walker et al., 2014) was used to polish the draft assembly with the filtered Illumina reads. The quality of gene prediction was evaluated using Benchmarking Universal Single-Copy Orthologs (BUSCO v5.3.2; *actinobacteria_phylum_odb10* dataset) (Manni et al., 2021). The prediction of protein-coding and RNA genes was performed using Prokka (v1.13) (Seemann, 2014) and RNAmmer (v1.2) (Lagesen et al., 2007), respectively. Visual representation of genomic features, including gene prediction was performed using Circos (v0.69-9) (Krzywinski et al., 2009). The assignment of Clusters of Orthologous Groups (COGs) for the predicted genes was conducted using eggNOG-mapper (Cantalapiedra et al., 2021).

### 2.3 Species identification

The 16S rRNA gene sequence (1513 bp) retrieved from the genome was analyzed using the EZBioCloud 16S database (Yoon et al., 2017). Genomic relatedness indices, including digital DNA–DNA hybridization (dDDH) and average nucleotide identity, were calculated using the recommended settings of the Genome-to-Genome Distance Calculator (GGDC v3.0) (Meier-Kolthoff et al., 2013; Meier-Kolthoff et al., 2022) and Orthologous Average Nucleotide Identity Tool (v1.40) (Lee et al., 2016), respectively. Genomic relatedness was visualized in a scatter plot using *ggplot2*, *ggExtra*, and *ggthemes* packages (Wickham, 2016; Arnold, 2021; Baker, 2022) in R (v4.1.2) (Team, 2021).

### 2.4 Expression and purification of peptide SHC-AMP

Based on the identification pipeline for potential AMPs, peptide KPP03845_1_03451 named SHC-AMP was selected and then amplified by PCR using DNA of strain KPP03845 as a template and the following set of primers that contain EcoRI and XhoI restriction sites for subsequent insertion into the pGEX-4T-1 plasmid (Addgene, Cambridge, MA): 3451F (5′-AAGAATTCGTGGGCTCTGTTATCAAG-3′) and 3451R (5′-AACTCGAGTTACTTCTTGTTACGGCG-3′). Expand High Fidelity enzyme mix (Roche Diagnostics, Mannheim, Germany) was added to the reaction. Amplification was performed with an initial denaturation step at 95°C for 5 min, followed by 25 cycles of denaturation at 94°C for 1 min, annealing at 60°C for 1 min, extension at 72°C for 1 min and 30 s, and a final extension at 72°C for 3 min. The amplified peptide SHC-AMP was cloned into the pGEX-4T-1 plasmid vector, and then the recombinant plasmid was transformed into *E. coli* strain BL21 (DE3) (DYNE BIO, Seoul, Korea). For protein expression, the cell was cultured in Luria–Bertani (LB) broth (Difco Laboratories, Detroit, Mich.) containing ampicillin at 37°C overnight in a shaking incubator at 200 rpm. Twenty milliliters of overnight culture was transferred into 2 L of LB broth containing 100 µg mL^−1^ of ampicillin. Isopropyl-β-D-1-thiogalactopyranoside (Sigma Chemical Co., St Louis, MO, USA) was added to the culture to a final concentration of 1 mM during the late log phase (OD_600 =_ 0.6–0.8). Then, the culture was shaken further for 16 h at 18°C. After harvesting by centrifugation at 6900 ×*g* for 20 min, the cells were resuspended in buffer A containing 20 mM Tris-HCl (pH 8.0) and 100 mM NaCl and then disrupted by sonication. Cell debris was removed by centrifugation at 9500 ×*g* and 4°C for 1 h. The supernatant was loaded onto a glutathione-Sepharose column (GE Healthcare) equilibrated with buffer A for purification. After the column was washed with buffer A, the protein binding to the column was eluted using elution buffer containing 20 mM Tris-HCl (pH 8.0) and 20 mM reduced glutathione (Sigma Chemical Co., St Louis, MO, USA). Eluted protein was cleaved with thrombin at 4°C for 16 h. The eluted fractions (flow through, wash with buffer A, GST-KPP03845_1_03451, GST-cleaved KPP03845_1_03451 and GST) were separated using the SDS-PAGE on 15% gel (data not shown). The protein expression was confirmed by the size of the SDS-PAGE gel. The expressed KPP03845_1_03451 protein (69.6 µg) was examined for antifungal activity against *F. oxysporum* f. sp. *lycopersici*. The KPP03845_1_03451 amino acid sequence was synthesized as ^1^MGSVIKKRRKRMAKKKHRKLLKRTRVQRRNKK^32^ (Peptron, Daejeon, Republic of Korea). The synthesized protein sequence was determined using a high-performance liquid chromatography (HPLC) system (SHIMADZU Prominence HPLC System) equipped with Shiseido capcell pak C18 (4.6 × 50 mm, 5 μm, 120 Å) and mass spectrometer (SHIMADZU LCMS-2020 system). Solvent A consisted of water containing 0.1% trifluoroacetic acid, and solvent B consisted of acetonitrile containing 0.1% trifluoroacetic acid. The HPLC analysis was conducted at a flow rate of 1 mL/min using a linear gradient elution of 3–10% B (2 min), 10–40% B (10 min) and 40–60% B (1 min). Synthesized protein was monitored at 220 nm.

### 2.5 Homology modeling of peptide SHC-AMP

Three-dimensional structure of the peptide, SHC-AMP, was modelled by using the SWISS-MODEL homology modeling server (https://swissmodel.expasy.org) (Waterhouse et al., 2018). The amino acid sequence of SHC-AMP was graphically represented by using iCn3D (Wang et al., 2020).

### 2.6 Determination of the minimum inhibitory concentration (MIC) of SHC-AMP for various plant pathogens

The MIC values of the synthesized peptide were evaluated against various plant pathogens using the modified CLSI protocol in a 96-well plate (Rex, 2008). Then, 25 µL of conidial suspensions (final concentration of 4 × 10^5^ spores mL^-1^) of *Aspergillus oryzae*, *Alternaria brassicicola*, *Botrytis cinerea*, *Colletotrichum orbiculare*, *F. oxysporum* f. sp. *lycopersici*, and *Rhizopus stolonifer* var. *stolonifer* or bacterial suspensions (final concentration of 4 × 10^5^ CFU mL^-1^) of *Erwinia carotovora* subsp. *atroseptica* BAA672, *E. carotovora* subsp. *carotovora* ATCC39048, *Pseudomonas syringae* pv. *tomato* DC3000, and *Xanthomonas campestris* pv. *vesicatoria* Ds1 were added to 96-well plates (SPL life Sciences, Pocheon, Korea), followed by the addition of 49 µL of distilled water. The growth medium contained 25 µL of 4 × PDB or 4 × LB for fungi and bacteria, respectively. Subsequently, the synthesized peptide was added to each well at final concentrations of 0.5–512 µg mL^-1^. MICs of synthesized peptide were determined as the lowest concentration that caused complete growth inhibition by visual examination after 48 h.

### 2.7 Bacterial growth suppression assay in *A. thaliana*

Bacterial growth of *Pst* DC3000 in response to SHC-AMP treatment was confirmed in Col-0 as described in the previously reported method with minor modifications (Tornero and Dangl, 2001). *Pst* DC3000 suspension (1 × 10^5^ CFU mL^-1^) was inoculated into 5-week-old *A. thaliana* leaves using needleless syringe, until the suspension is spread to whole leaf. A series of concentrations of SHC-AMP (32 μg, 64 μg, and 128 μg mL^-1^) were infiltrated 1 day post-*Pst* DC3000 infection in the same way with *Pst* DC3000. Two and four days after bacterial infection, four leaf discs (6 mm in diameter) were collected for each sampling with three replicates to measure bacterial growth. In case of 0 dpi, leaves were collected after water soaking area disappeared. Leaf discs were ground in homogenizer with 300 μl of 10 mM of MgCl_2_. 700 μl of distilled water was added to meet 1 mL, and ten-fold serial dilution was performed with distilled water. Diluted samples (10 μl) were spotted on King’s B agar plate supplemented with rifampicin and cycloheximide at a final concentration of 25 μg mL^-1^. After culturing bacteria in 28°C incubator overnight, the number of CFU was counted for each sample. Disease damage was calculated as damaged leaf area/total leaf area × 100%. The effect of SHC-AMP treatment in *Pst* DC3000 infected plant leaves was analyzed by one-way analysis of variance (ANOVA) using SAS 9.4 (SAS Inst., Cary, NC, USA). When ANOVA indicated significance, means were separated using Least Significant Difference (LSD) test at α = 0.05.

## 3 Results

### 3.1 Identification and distribution of highly conserved proteins in the genus *Streptomyces*

**Figure 1:**
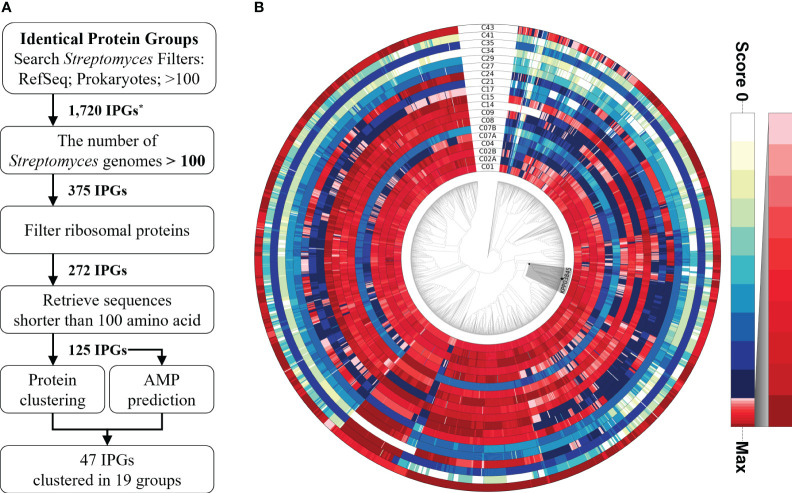
Identification pipeline for potential antimicrobial peptides among highly conserved proteins in the genus *Streptomyces*. **(A)** A flowchart of the pipeline to search for a potential antimicrobial peptide from the highly conserved proteins in *Streptomyces* spp. **(B)** Relative ratio to the maximum homology score for each sequence profile is shown in the color gradient on the right. A monophyletic group, including the strain KPP03845 and 105 *Streptomyces* spp., is shaded in gray. The strain KPP03845 is indicated with a filled star mark at the terminal node. ^*^IPG, Identical Protein Group.

An identification pipeline was constructed to search for potential antimicrobial proteins that are highly conserved in the genus *Streptomyces*. The NCBI IPG database was used as the initial database to identify candidate sequences. A total of 1720 protein sequences were found in more than 100 prokaryotic genomes, including those belonging to *Streptomyces* in the RefSeq database. Of these protein sequences, only 375 were found in more than 100 *Streptomyces* genomes, yielding 272 IPGs after filtering out ribosomal proteins. Ribosomal proteins were identified on the basis of protein annotation from the NCBI Gene database and discarded because of their conserved structure and function during evolution (Timsit et al., 2021). Only sequences shorter than 100 amino acids (aa) in length were retained for further analysis because the majority (97.54% or 3089/3167) of AMPs in the Antimicrobial Peptide Database (APD3; https://aps.unmc.edu/downloads; last accessed on July 18, 2022) (Wang et al., 2016) were shorter than 100 aa in length. AMP prediction was performed using three recently developed tools, resulting in 125 sequences (Veltri et al., 2018; Pinacho-Castellanos et al., 2021; Pirtskhalava et al., 2021). Of the 125 sequences, 47 were predicted to be AMPs by at least one predictor, 11 sequences by two predictors, and one sequence by all three (**Supplementary Table 4**). The 47 proteins belonged to 17 clusters (19 subclusters) based on protein clustering analysis of the 125 sequences. Subsequently, HMM sequence profiles built for the 19 subclusters were used to scan 2062 *Streptomyces* genomes and 762 selected Actinobacterial genomes. The homology distribution showed intricate patterns, showing wide degrees of sequence conservation (**Figure 1**, **Supplementary Figure 1**). Only few genes encoding highly homologous proteins were functionally characterized, playing regulatory roles in sporulation, morphology, and antibiotic production (**Supplementary Table 5**). Cluster43, representing a SHC-AMP, was selected for experimental validation because significant sequence similarities were found in 2054 out of 2062 *Streptomyces* genomes, and all of the three tools were predicted it to be an AMP.

### 3.2 Species identification and genome summary for the strain KPP03845

Several reports have been found novel AMPs identified in *Streptomyces* strains, all of which exhibited strong antimicrobial activity (Vasilchenko et al., 2020; Zhang et al., 2020). Meanwhile, *Streptomyces* strain KPP03845 was isolated from soil, and its high degree of antifungal activity against plant pathogenic fungi was demonstrated (**Supplementary Figure 2**). The culture filtrate (2 mL) of strain KPP03845 showed inhibition zone diameters of 35 mm, 25 mm, 28 mm, 24 mm, 30 mm and 32 mm for *A. brassicicola*, *A. oryzae*, *C. gloeosporioides*, *C. orbiculare*, *F. oxysporum* f. sp. *lycopersici* and *F. oxysporum* f. sp. *lycopersici*, respectively. The culture filtrate did not show a clear inhibition zone against *R. stolonifer* var. *stolonifera*. Thus, the strain KPP03845 was sequenced for use in comparative genome analysis and as a genetic template for heterologous expression and peptide synthesis.

**Figure 2:**
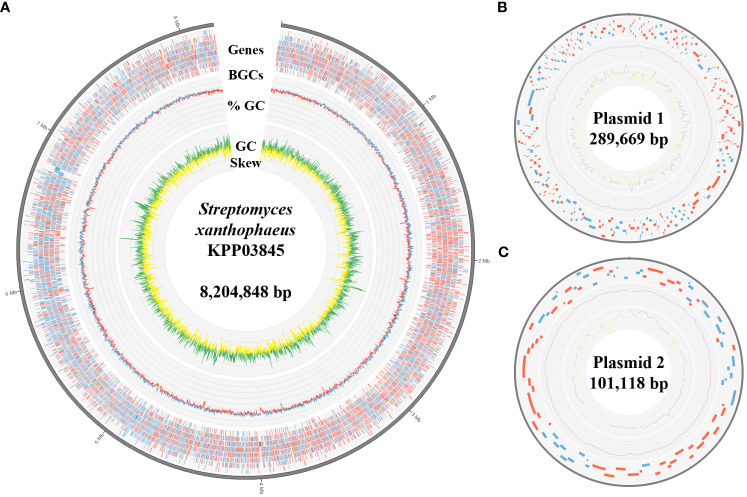
Genomic features of S. xanthophaeus strain KPP03845. **(A)** The circular diagram represents the chromosome of the strain KPP03845. From the outermost track to the center: (i) predicted genes (blue/red for forward/reverse strand), (ii) GC content (blue/red for above/below average), and (iii) GC skew (green: >0 and yellow: <0). **(B, C)** Circular diagrams of the two plasmids. Tracks from the outside show the predicted genes, GC content, and GC skew.

The complete genome of the strain KPP03845 was assembled in a single chromosome of 8204848 bp with 72.31% GC content. In addition, two plasmids were assembled into molecules of 289669 and 101118 bp (**Table 1**, **Figure 2**). A total of 6262 protein-coding genes were assigned with COGs, yielding 7018 hits (**Supplementary Table 6**). In addition to gene matches with unknown functions, the top five largest groups included genes involved in transcription (859), amino acid transport and metabolism (680), signal transduction mechanism (518), carbohydrate transport and metabolism (487), and inorganic ion transport and metabolism (390) (**Supplementary Table 6**).

**Table 1:** Genomic features of *Streptomyces xanthophaeus* strain KPP03845.

Genomic Feature	Value
Size of the genome assembly (bp)	Chromosome: 8,204,848 Plasmids: 289,669/101,118
GC content (%)	70.29
Protein-coding genes/regions (bp)/Avg. CDS (aa)	7,750/7,187,586/322
tRNA/rRNA genes	88/21
The number of genes assigned to COG categories	7,372
Complete BUSCOs (%)	100

The 16S rRNA gene of the strain KPP03845 (1513 bp) showed 100% similarity spanning 1447 bp with that of *Streptomyces xanthophaeus* NRRL B-5414^T^, *S. nojiriensis* LMG 20094^T^, and *S. spororaveus* LMG 20313^T^. In addition, 11 other strains had similarity greater than 99%, hindering accurate species identification (**Supplementary Table 7**). Indices of genomic relatedness, dDDH and OrthoANI, were calculated for 2061 *Streptomyces* genomes (**Supplementary Table 2**) to identify the KPP03845 strain. The widely accepted criteria for species demarcation (Auch et al., 2010; Lee et al., 2016) were applied to the calculations. Two *S. xanthophaeus* genomes with OrthoANI values greater than 98% and dDDH values of 83.5% were identified (**Supplementary Figure 3**, **Supplementary Table 2**). Collectively, the strain KPP03845 was identified as *S. xanthophaeus*.

### 3.3 Production and purification of peptide SHC-AMP

**Figure 3:**
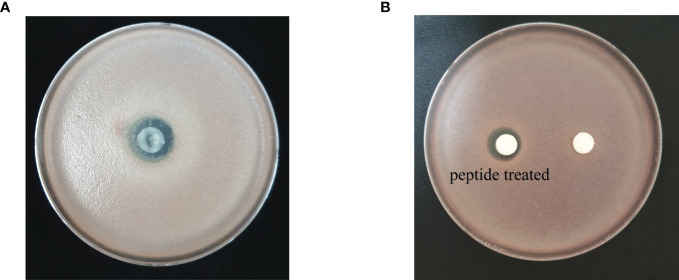
Antifungal activity of **(A)** recombinant peptide SHC-AMP and **(B)** synthesized peptide SHC-AMP against *Fusarium oxysporum* f. sp. *lycopersici*.

Based on the prediction results from the identification pipeline, the KPP03845_1_03451 gene was selected to test for antimicrobial activity. The full-length open reading frame of KPP03845_1_03451 (M1 to K32) from the gDNA of *S*. *xanthophaeus* strain KPP03845 was cloned into the expression vector, pGEX-4T-1, between the EcoRI and XhoI restriction enzyme sites. Then, the recombinant protein was expressed in *E. coli* BL21 (DE3). After the purification of the protein by affinity chromatography using glutathione-Sepharose, 69.6 µg of the purified protein was shown to have antifungal activity against *F. oxysporum* f. sp. *lycopersici* (**Figure 3**). In addition, 300 µg of the synthesized peptide SHC-AMP exhibited antifungal activity against *F. oxysporum* f. sp. *lycopersici* (**Figure 3**).

### 3.4 Antimicrobial activity of the synthesized peptide SHC-AMP on various plant pathogens

The antimicrobial activity of the synthesized SHC-AMP was evaluated against various plant pathogens by using modified CLSI microdilution method. The synthesized peptide exhibited antimicrobial activity against several plant pathogenic bacteria and fungi used in this assay (**Table 2**). The growth of *X. campestris* pv. *vesicatoria* Ds1 and *P. syringae* pv. *tomato* DC3000 was inhibited at 256 and 128 µg mL^-1^, respectively, whereas the antibacterial activity against *E. carotovora* subsp. *atroseptica* BAA672 and *E. carotovora* subsp. *carotovora* was not observed. The synthesized peptide inhibited the mycelial growth of *A. brassicicola*, *B. cinerea*, and *F*. *oxysporum* f. sp. *lycopersici* at concentrations of 32 to 128 µg mL^-1^, whereas the mycelial growth of *A. oryzae*, *C. orbiculare*, or *R. stolonifer* var. *stolonifer* was not inhibited even at a concentration of 512 µg mL^-1^.

**Table 2:** Minimum inhibitory concentrations (MICs) of the synthesized peptide SHC-AMP against various plant pathogens.

Plant pathogenic microorganism	MICs^a^ (µg mL^-1^)
	SHC-AMP
*Erwinia carotovora* subsp. *atroseptica* BAA672	>512^b^
*Erwinia carotovora* subsp. *carotovora* ATCC39048	>512^b^
*Pseudomonas syringae* pv. *tomato* DC3000	128
*Xanthomonas campestris* pv. *vesicatoria* Ds1	256
*Aspergillus oryzae*	>512^b^
*Alternaria brassicicola*	128
*Botrytis cinerea*	128
*Colletotrichum orbicular*	>512^b^
*Fusarium oxysporum* f.sp. *lycopersici*	32
*Rhizopus stolonifer* var. *stolonifer*	>512^b^

a The lowest concentration that completely inhibited the growth of the plant pathogens was determined after incubation for 48 h.

b The value of >512 represents that growth of the test plant pathogenic microorganism was not inhibited at concentrations up to 512 µg mL^-1^.

### 3.5 Growth suppression against *Pseudomonas syringae* pv. *tomato* DC3000

**Figure 4:**
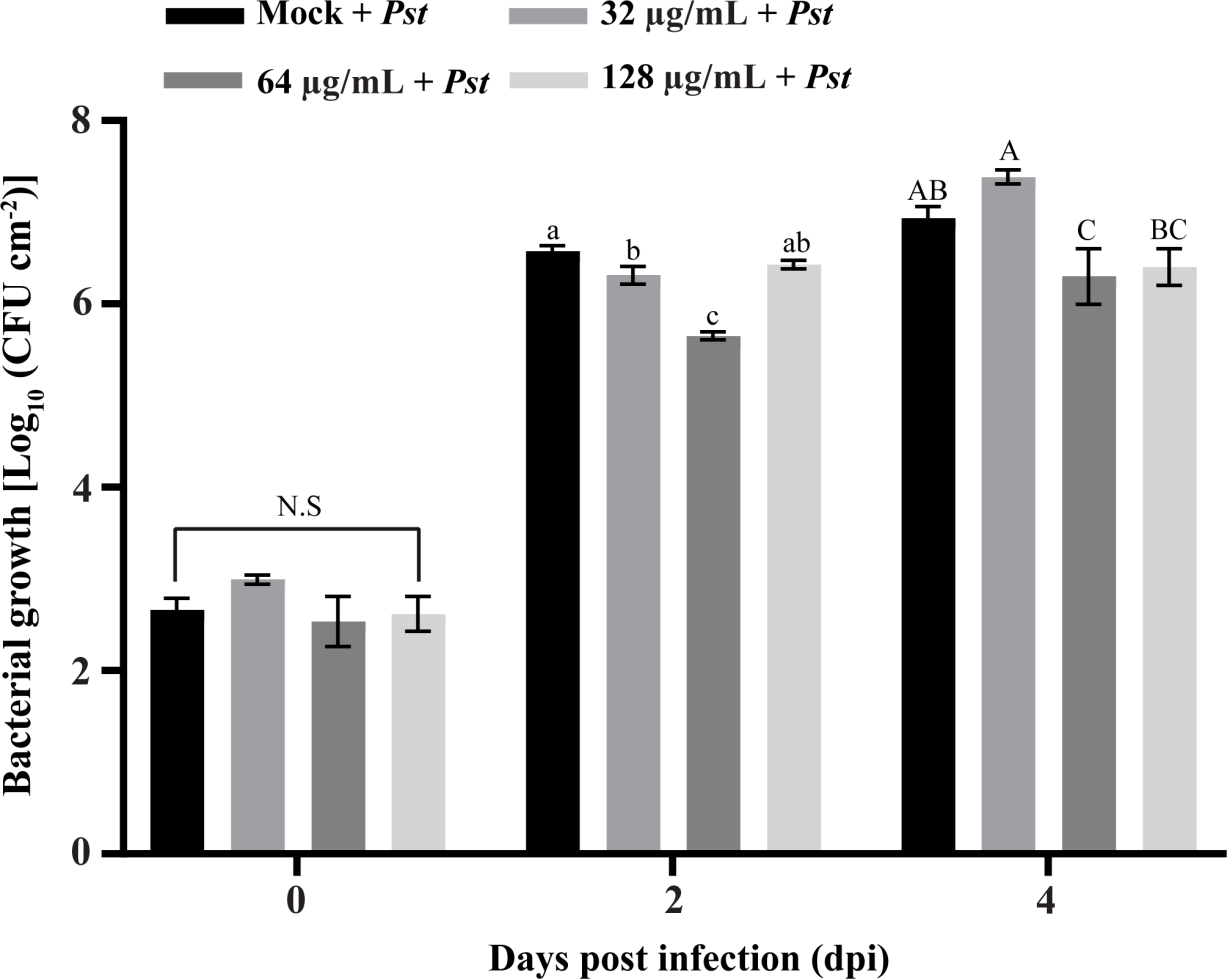
Biocontrol ability of SHC-AMP against *Pseudomonas syringae* pv. *tomato* DC3000 infection in *Arabidopsis thaliana*. The “Mock + *Pst*” group means inoculated with *Pst* DC3000 suspension, then treated with water instead of SHC-AMP. 32 μg, 64 μg, 128 μg mL^-1^ of SHC-AMP treated 1 day post-*Pst* DC3000 infection. Mean separation followed analysis of variance (ANOVA) with LSD test at α = 0.05. Means followed by different letters indicate a significant difference between SHC-AMP-untreated and SHC-AMP-treated plants with different concentrations on each day.

The synthesized SHC-AMP was evaluated with regard to its ability to suppress *Pst* DC3000 infection in *A. thaliana* leaves. The symptoms developed at 2 days after bacterial infection in the SHC-AMP-untreated control. Four days after infection, *A. thaliana* leaves treated with the SHC-AMP at a concentration of 64 µg mL^-1^ exhibited attenuated *Pst* DC3000-infected symptoms compared to the untreated control (**Supplementary Figure 4**). However, *A. thaliana* leaves treated with the 32 µg mL^-1^ and 128 µg mL^-1^ were observed to have similar symptoms of chlorosis and necrosis to the control leaves at 4 days after infection. *A. thaliana* leaves treated with the 0 µg mL^-1^, 32 µg mL^-1^, 64 µg mL^-1^, and 128 µg mL^-1^ exhibited infection damage of 36.07%, 29.38%, 1.21%, and 26.59%, respectively. For further confirmation of the reduced *Pst* DC3000 growth in the SHC-AMP-treated *A. thaliana* leaves, the number of bacterial colonies grown *in planta* was monitored (**Figure 4**). The number of colonies in the SHC-AMP-untreated leaves were observed to be 39 × 10^5^ colony-forming units (CFU)/cm^2^ and 93 × 10^5^ CFU/cm^2^ on average at 2 days and 4 days after inoculation of *Pst* DC3000. Compared to the SHC-AMP-untreated control at 2 and 4 days after inoculation, bacterial growth was reduced in leaves treated with 64 µg mL^-1^ of the peptide. Treatment with 64 µg mL^-1^ of the SHC-AMP reduced the bacterial growth of *Pst* DC3000 by 8.54 and 2.82 times compared to control, respectively. Initially, treatment with 32 µg mL^-1^ of the SHC-AMP suppressed the growth of *Pst* DC3000, but suppression of bacterial growth was not observed at 4 days after infection. The treatment with 128 µg mL^-1^ of the SHC-AMP resulted in a decrease in bacterial growth until 4 days after inoculation, but statistically significant results were not obtained. Thus, the most effective concentration of SHC-AMP was determined to be 64 µg mL^-1^ with less necrotic symptoms.

## 4 Discussion

A surge in the number of sequenced genomes in the last decade promoted the exploration of the genetic diversity of the genus *Streptomyces*. Although it is the most heavily sequenced genera in the phylum Actinobacteria, the roles of highly conserved genes in this genus have been poorly investigated (Gao and Gupta, 2012; Zhou et al., 2012). In this study, the culture filtrate of *S. xanthophaeus* strain KPP03845 had inhibitory effects on the mycelial growth of *A. oryzae*, *A. brassicicola*, *C. orbiculare*, *C. gloeosporioides*, *F. oxysporum* f. sp. *cucumerinum*, *F*. *oxysporum* f. sp. *lycopersici*, and *R. stolonifer* var. *stolonifera* (**Supplementary Figure 2**). Based on previous reports, a large variety of oligo- and polypeptides with antimicrobial activity have been produced by the genus *Streptomyces* (Bockus et al., 2013). Therefore, *S. xanthophaeus* strain KPP03845 was selected as a good candidate source for the isolation of AMPs and conserved proteins in this genus.

A total of 272 protein sequences highly conserved in *Streptomyces* were considered as good candidates for generating a target gene pool with putative antimicrobial activity. A total of 125 protein sequences highly conserved in the genus *Streptomyces* were identified using the NCBI IPG database (**Figure 1**). Subsequently, 19 DNA sequence profiles predicted to encode AMPs were matched against 2061 *Streptomyces* genomes (**Figure 1**). To date, only a few of the predicted peptides have been functionally characterized to date, which play regulatory roles in morphology and antibiotic production (**Supplementary Table 5**). Genome analysis and three prediction tools (Veltri et al., 2018; Pinacho-Castellanos et al., 2021; Pirtskhalava et al., 2021) suggested that the highly conserved peptide, KPP03845_1_03451 (SHC-AMP), found in 2054 out of 2062 genomes, may have antimicrobial activity (**Supplementary Table 4**). This 32-aa peptide, is unusually rich in basic amino acids, including arginine (25.00%) and lysine (31.25%) (**Supplementary Figure 5**). Arginine-rich peptides, in combination with other amino acids (lysine, serine, proline, glycine, tryptophan, valine, and glutamic acid), can function as cellular pathway regulators and potent antimicrobial agents (Chandana and Venkatesh, 2016). These peptides have the innate ability to combat pathogenic invasions (Radek and Gallo, 2007). In general, they comprised <100 amino acids with positively charged and hydrophobic amino acids. Given these characteristics, these cationic peptides exhibited potential antibacterial and antifungal activities because they can bind to negatively charged phospholipid membranes and penetrate the cytoplasmic matrix (Radek and Gallo, 2007). For example, arginine-rich protegrins exhibit potential antibacterial and antifungal activities. However, the function of the peptide SHC-AMP remains unknown; only its expression pattern in nitrogen-restricted medium has been reported (Gidalevitz et al., 2003; Lewis et al., 2011). Thus, it might be optimized to work only in a given composition and environment. We confirmed that the peptide SHC-AMP has a broad spectrum of antimicrobial activity against agronomically important plant pathogenic bacteria and fungi (**Table 2**). The munumbicins A, B, C, and D produced by *Streptomyces* sp. NRRL 30562 have been reported as wide-spectrum antibiotics against plant pathogens including *Geotrichum candidum*, *Phytophthora cinnamomi*, *Pseudomonas syringae*, *Pythium ultimum*, *Rhizoctonia solani*, and *Sclerotinia sclerotiorum* (Castillo et al., 2002). The MIC values of these peptides against plant pathogens ranged from 0.2 to 31.2 µg mL^-1^. Collectively, these results indicate that the ability of Actinomycetes to protect themselves from invading microorganisms or other species may be a key factor in survival during evolution. Although its exact role remains unclear, the major role of peptide SHC-AMP would be modulation of the immune system to control and limit microbial infection.

The synthesized SHC-AMP was tested for its ability to protect *A. thaliana* against infection by *Pst* DC3000 *in vivo* (**Figure 4**, **Supplementary Figure 4**). The peptide can effectively protect *A. thaliana* against *Pst* DC3000 infection after treatment at 64 µg mL^-1^. Recently, the arginine-rich SM-985 peptide has been reported to inhibit protection against leaf spot disease infection caused by *Pst* DC3000 (Qutb et al., 2020). Cathelicidins, such as proline/arginine peptides, have shown activity against antibiotic-resistant bacteria such as methicillin-resistant *Staphylococcus aureus* (Turner et al., 1998), vancomycin-resistant *Enterococcus faecalis* (Skerlavaj et al., 1999), and multi-resistant *Pseudomonas aeruginosa* (Turner et al., 1998). These results indicate that the SHC-AMP can used as major ingredient in pesticide for plant disease control. However, the effect of SHC-AMP was reduced at the concentration of 128 µg mL^-1^. The disease control efficacy of antimicrobial agents on pathogen-infected plants is related to their concentration, but it is not always positively correlated. Our results suggested that the most effective concentration of SHC-AMP against *Pst* DC3000 infection in *A. thaliana* is at the concentration of 64 µg mL^-1^.

AMP analyses of 2061 *Streptomyces* genomes have led to the discovery of 19 highly conserved genes that have antimicrobial characteristics. These peptides will provide novel targets for agricultural and biotechnological applications. The present results show the antifungal efficacy of the SHC-AMP against plant pathogens and its specific ability to protect *A. thaliana* from infection by *Pst* DC3000. Thus, the highly conserved peptide, SHC-AMP, was considered as a newly defined AMP that inhibits the growth of plant pathogens.

## 5 Conclusion

Comparative analysis of 2061 *Streptomyces* genomes with the NCBI IPG database led to the identification of 19 sequences as potential AMPs that are highly conserved in the genus *Streptomyces*. The KPP03845 strain was phylogenomically identified as a member of *S. xanthophaeus*. The peptide SHC-AMP was one of the 19 highly conserved peptides across the genus *Streptomyces*. The SHC-AMP exhibited antimicrobial activities against bacterial and fungal pathogens. In particular, the SHC-AMP can also protect *A. thaliana* against *Pst* DC3000 infection. A new AMP was discovered from the genome sequence of the strain KPP03845 by the comparative genome analysis which provided an outlook of highly conserved proteins in the genus *Streptomyces*.

## Data availability statement

The datasets presented in this study can be found in online repositories. The names of the repository/repositories and accession number(s) can be found in the article/**Supplementary Material**.

## Author contributions

Formal analysis, investigation, BJ, NY, and JC. Conceptualization, methodology, BJ and JC. Resources, BJ. Software, data curation, visualization, JC. Writing original draft, writing review & editing, BJ, JK, and JC. Funding acquisition, JK. All authors contributed to the article and approved the submitted version.
