# Supplementary material for: A peptide encoded by a highly conserved gene belonging to the genus Streptomyces shows antimicrobial activity against plant pathogens
Source: Front Plant Sci. 2023 Oct 5;14:1250906. doi: 10.3389/fpls.2023.1250906 (PMC10585065; doi:10.3389/fpls.2023.1250906)
Supplement: Supplementary file 2 [file DataSheet_1.pdf]

Supplementary Material

A peptide encoded by a highly conserved gene belonging to the genus *Streptomyces* shows antimicrobial activity against plant pathogens

Byeong Jun Jeon[1], Nayeon Yoo[2], Jeong Do Kim[1,*], and Jaeyoung Choi[3,*]

[1]Smart Farm Research Center, Korea Institute of Science and Technology, Gangneung 25451, Republic of Korea

[2]Department of Plant Biotechnology, Korea University, Seoul 02481, Republic of Korea

[3]Department of Oriental Medicine Biotechnology, College of Life Sciences, Kyung Hee University, Yongin 17104, Republic of Korea

[*] **Correspondence:**

Jeong Do Kim

kimjeongdo@kist.re.kr

Jaeyoung Choi

jaeyoung.choi@khu.ac.kr

Supplementary Tables

Table S1. List of 47 clusters obtained from mcl analysis of 125 IPG sequences (provided as a separate file)

Table S2. Genomic relatedness between the strain KPP03845 and each of the 2,061 *Streptomyces* spp. (provided as a separate file)

Table S3. List of 761 actinobacterial genomes used in the analysis (provided as a separate file)

Table S4. Antimicrobial peptide prediction results of the 125 IPG sequences by Antimicrobial Peptide Scanner (v2), a prediction tool implemented in database of antimicrobial activity and structure of peptides (DBAASP v3.0), and AMPDiscover

Name	Cluster	1*	2*	3*	4*
WP_003948780.1	Cluster01	Non-AMP	Non-AMP	AMP	1
WP_003974443.1	Cluster01	Non-AMP	Non-AMP	AMP	1
WP_003967102.1	Cluster01	Non-AMP	Non-AMP	AMP	1
WP_003967346.1	Cluster01	Non-AMP	Non-AMP	Non-AMP	0
WP_003986833.1	Cluster01	Non-AMP	Non-AMP	Non-AMP	0
WP_005315736.1	Cluster01	Non-AMP	Non-AMP	Non-AMP	0
WP_003949956.1	Cluster01	Non-AMP	Non-AMP	Non-AMP	0
WP_003978336.1	Cluster01	Non-AMP	Non-AMP	Non-AMP	0
WP_004984723.1	Cluster01	Non-AMP	Non-AMP	Non-AMP	0
WP_004929928.1	Cluster01	Non-AMP	Non-AMP	Non-AMP	0
WP_003984261.1	Cluster01	Non-AMP	Non-AMP	Non-AMP	0
WP_015607926.1	Cluster01	Non-AMP	Non-AMP	Non-AMP	0
WP_003992177.1	Cluster01	Non-AMP	Non-AMP	Non-AMP	0
WP_003964323.1	Cluster01	Non-AMP	Non-AMP	Non-AMP	0
WP_004986573.1	Cluster01	Non-AMP	Non-AMP	Non-AMP	0
WP_007383480.1	Cluster01	Non-AMP	Non-AMP	Non-AMP	0
WP_003969786.1	Cluster01	Non-AMP	Non-AMP	Non-AMP	0
WP_003947520.1	Cluster01	Non-AMP	Non-AMP	Non-AMP	0
WP_003975777.1	Cluster02A	Non-AMP	Non-AMP	AMP	1
WP_004952403.1	Cluster02A	Non-AMP	Non-AMP	AMP	1
WP_003983763.1	Cluster02A	Non-AMP	Non-AMP	AMP	1
WP_014046372.1	Cluster02A	Non-AMP	Non-AMP	AMP	1
WP_003950432.1	Cluster02A	Non-AMP	Non-AMP	AMP	1
WP_003968728.1	Cluster02A	Non-AMP	Non-AMP	AMP	1
WP_003992873.1	Cluster02B	AMP	Non-AMP	AMP	2
WP_003950777.1	Cluster02B	AMP	Non-AMP	AMP	2
WP_016639615.1	Cluster02B	AMP	Non-AMP	AMP	2
WP_003953983.1	Cluster02B	AMP	Non-AMP	AMP	2
WP_003973730.1	Cluster02B	AMP	Non-AMP	AMP	2
WP_003983230.1	Cluster02B	AMP	Non-AMP	AMP	2
WP_004937597.1	Cluster02B	Non-AMP	Non-AMP	AMP	1
WP_003988945.1	Cluster03	Non-AMP	Non-AMP	Non-AMP	0

WP_003982715.1	Cluster03	Non-AMP	Non-AMP	Non-AMP	0
WP_003970369.1	Cluster03	Non-AMP	Non-AMP	Non-AMP	0
WP_005319902.1	Cluster03	Non-AMP	Non-AMP	Non-AMP	0
WP_003977348.1	Cluster03	Non-AMP	Non-AMP	Non-AMP	0
WP_004948662.1	Cluster03	Non-AMP	Non-AMP	Non-AMP	0
WP_003965959.1	Cluster04	Non-AMP	AMP	Non-AMP	1
WP_005311361.1	Cluster04	Non-AMP	AMP	Non-AMP	1
WP_003980229.1	Cluster04	Non-AMP	AMP	Non-AMP	1
WP_003973401.1	Cluster04	Non-AMP	AMP	Non-AMP	1
WP_005479813.1	Cluster04	Non-AMP	AMP	Non-AMP	1
WP_003951123.1	Cluster04	Non-AMP	Non-AMP	Non-AMP	0
WP_003951011.1	Cluster05	Non-AMP	Non-AMP	Non-AMP	0
WP_003966094.1	Cluster05	Non-AMP	Non-AMP	Non-AMP	0
WP_003973500.1	Cluster05	Non-AMP	Non-AMP	Non-AMP	0
WP_015036350.1	Cluster05	Non-AMP	Non-AMP	Non-AMP	0
WP_006130840.1	Cluster05	Non-AMP	Non-AMP	Non-AMP	0
WP_003955420.1	Cluster06	Non-AMP	Non-AMP	Non-AMP	0
WP_003949502.1	Cluster06	Non-AMP	Non-AMP	Non-AMP	0
WP_003999914.1	Cluster06	Non-AMP	Non-AMP	Non-AMP	0
WP_003967677.1	Cluster06	Non-AMP	Non-AMP	Non-AMP	0
WP_004943146.1	Cluster06	Non-AMP	Non-AMP	Non-AMP	0
WP_003970432.1	Cluster07A	AMP	Non-AMP	AMP	2
WP_003946963.1	Cluster07A	AMP	Non-AMP	AMP	2
WP_003977410.1	Cluster07A	Non-AMP	Non-AMP	AMP	1
WP_003950656.1	Cluster07B	AMP	Non-AMP	Non-AMP	1
WP_003948050.1	Cluster07B	Non-AMP	Non-AMP	Non-AMP	0
WP_003965732.1	Cluster08	Non-AMP	Non-AMP	AMP	1
WP_005481602.1	Cluster08	Non-AMP	Non-AMP	AMP	1
WP_003951337.1	Cluster08	Non-AMP	Non-AMP	AMP	1
WP_003993510.1	Cluster08	Non-AMP	Non-AMP	AMP	1
WP_003990598.1	Cluster09	AMP	Non-AMP	Non-AMP	1
WP_003950507.1	Cluster09	Non-AMP	Non-AMP	Non-AMP	0
WP_003968811.1	Cluster09	Non-AMP	Non-AMP	Non-AMP	0
WP_007382399.1	Cluster09	Non-AMP	Non-AMP	Non-AMP	0
WP_006123601.1	Cluster10	Non-AMP	Non-AMP	Non-AMP	0
WP_003976983.1	Cluster10	Non-AMP	Non-AMP	Non-AMP	0
WP_004002281.1	Cluster10	Non-AMP	Non-AMP	Non-AMP	0
WP_003947402.1	Cluster10	Non-AMP	Non-AMP	Non-AMP	0
WP_003948727.1	Cluster11	Non-AMP	Non-AMP	Non-AMP	0
WP_003974374.1	Cluster11	Non-AMP	Non-AMP	Non-AMP	0
WP_004927567.1	Cluster11	Non-AMP	Non-AMP	Non-AMP	0
WP_004986406.1	Cluster12	Non-AMP	Non-AMP	Non-AMP	0
WP_003949043.1	Cluster12	Non-AMP	Non-AMP	Non-AMP	0
WP_003968135.1	Cluster12	Non-AMP	Non-AMP	Non-AMP	0
WP_010058136.1	Cluster13A	Non-AMP	Non-AMP	Non-AMP	0
WP_003947485.1	Cluster13A	Non-AMP	Non-AMP	Non-AMP	0
WP_008410190.1	Cluster13B	Non-AMP	Non-AMP	Non-AMP	0
WP_003976229.1	Cluster14	AMP	Non-AMP	Non-AMP	1
WP_006136074.1	Cluster14	AMP	Non-AMP	Non-AMP	1
WP_003969224.1	Cluster14	AMP	Non-AMP	Non-AMP	1
WP_003956442.1	Cluster15	Non-AMP	AMP	Non-AMP	1
WP_003948620.1	Cluster15	Non-AMP	AMP	Non-AMP	1

WP_004927214.1	Cluster15	Non-AMP	AMP	Non-AMP	1
WP_003966321.1	Cluster16	Non-AMP	Non-AMP	Non-AMP	0
WP_003961784.1	Cluster16	Non-AMP	Non-AMP	Non-AMP	0
WP_003992906.1	Cluster16	Non-AMP	Non-AMP	Non-AMP	0
WP_006123846.1	Cluster17	AMP	Non-AMP	Non-AMP	1
WP_003947575.1	Cluster17	AMP	Non-AMP	Non-AMP	1
WP_003947636.1	Cluster18	Non-AMP	Non-AMP	Non-AMP	0
WP_003969641.1	Cluster18	Non-AMP	Non-AMP	Non-AMP	0
WP_003997603.1	Cluster19	Non-AMP	Non-AMP	Non-AMP	0
WP_006127869.1	Cluster19	Non-AMP	Non-AMP	Non-AMP	0
WP_003948191.1	Cluster20	Non-AMP	Non-AMP	Non-AMP	0
WP_003968990.1	Cluster20	Non-AMP	Non-AMP	Non-AMP	0
WP_003947634.1	Cluster21	Non-AMP	Non-AMP	AMP	1
WP_003969643.1	Cluster21	Non-AMP	Non-AMP	AMP	1
WP_003973418.1	Cluster22	Non-AMP	Non-AMP	Non-AMP	0
WP_003951107.1	Cluster22	Non-AMP	Non-AMP	Non-AMP	0
WP_005485166.1	Cluster23	Non-AMP	Non-AMP	Non-AMP	0
WP_003947232.1	Cluster23	Non-AMP	Non-AMP	Non-AMP	0
WP_003975360.1	Cluster24	Non-AMP	Non-AMP	AMP	1
WP_003967454.1	Cluster24	Non-AMP	Non-AMP	Non-AMP	0
WP_003999697.1	Cluster25	Non-AMP	Non-AMP	Non-AMP	0
WP_003958712.1	Cluster25	Non-AMP	Non-AMP	Non-AMP	0
WP_004002138.1	Cluster26	Non-AMP	Non-AMP	Non-AMP	0
WP_003948664.1	Cluster27	AMP	AMP	Non-AMP	2
WP_003946983.1	Cluster28	Non-AMP	Non-AMP	Non-AMP	0
WP_003948677.1	Cluster29	AMP	Non-AMP	AMP	2
WP_008409915.1	Cluster30	Non-AMP	Non-AMP	Non-AMP	0
WP_008409760.1	Cluster31	Non-AMP	Non-AMP	Non-AMP	0
WP_008409756.1	Cluster32	Non-AMP	Non-AMP	Non-AMP	0
WP_003950786.1	Cluster33	Non-AMP	Non-AMP	Non-AMP	0
WP_003969918.1	Cluster34	Non-AMP	AMP	Non-AMP	1
WP_003951302.1	Cluster35	Non-AMP	Non-AMP	AMP	1
WP_003947204.1	Cluster36	Non-AMP	Non-AMP	Non-AMP	0
WP_004984898.1	Cluster37	Non-AMP	Non-AMP	Non-AMP	0
WP_006127526.1	Cluster38	Non-AMP	Non-AMP	Non-AMP	0
WP_003949541.1	Cluster39	Non-AMP	Non-AMP	Non-AMP	0
WP_026048345.1	Cluster40	Non-AMP	Non-AMP	Non-AMP	0
WP_008405281.1	Cluster41	AMP	Non-AMP	AMP	2
WP_003966491.1	Cluster42	Non-AMP	Non-AMP	Non-AMP	0
**WP_003948845.1**	**Cluster43**	**AMP**	**AMP**	**AMP**	**3**
WP_016434100.1	Cluster44	Non-AMP	Non-AMP	Non-AMP	0

*1: Antimicrobial Peptide Scanner (v2), **2**: a prediction tool implemented in database of antimicrobial activity and structure of peptides (DBAASP v3.0), **3**: AMPDiscover, and **4**: the number of positive predictions.

Table S5. Characterized genes of the 19 antimicrobial peptide candidates that are highly conserved in the genus *Streptomyces*

Matching Cluster	SCO locus*	Gene symbol	Function	Species	Ref.

Cluster01	SCO4505				
Cluster02A	SCO3034	*whiB*	essential for sporulation	*Streptomyces coelicolor* A3(2)	(Davis and Chater, 1992)
Cluster02B	SCO5240				
Cluster04	SCO5592				
Cluster07A	SCO1415	*smeA*	defects in chromosome segregation; heterogeneous spore sizes; reduced heat resistance	*Streptomyces coelicolor* M145	(Ausmees et al., 2007)
Cluster07B	SCO5123				
Cluster08	SCO5864				
Cluster09	SCO2950				
Cluster14	SCO2574				
Cluster15	SCO4725				
Cluster17	SCO2078	*sepG*	compromised in sporulation; spore-wall synthesis andnucleoid morphology	*S. coelicolor* M145	(Zhang et al., 2016)
Cluster21	SCO2138				
Cluster24	SCO3576				
Cluster27	SCO4646				
Cluster29	SCO4613				
Cluster34	SCO4797				
Cluster35	N/A				
Cluster41	SCO6509				
Cluster43	SCO3327				

*In order to retrieve SCO locus names, consensus sequences of each cluster were searched against in the *Streptomyces coelicolor* A3(2) proteome obtained from the FTP server of Wellcome Sange Institute (ftp://ftp.sanger.ac.uk/pub/project/pathogens/S_coelicolor/).

Table S6. Functional annotation of the predicted proteins in the strain KPP03845

COG*	Description	Hits
A	RNA processing and modification	4
B	Chromatin structure and dynamics	3
C	Energy production and conversion	377
D	Cell cycle control, cell division, chromosome partitioning	88
E	Amino acid transport and metabolism	680
F	Nucleotide transport and metabolism	149
G	Carbohydrate transport and metabolism	487
H	Coenzyme transport and metabolism	227
I	Lipid transport and metabolism	356
J	Translation, ribosomal structure and biogenesis	252
K	Transcription	859
L	Replication, recombination and repair	327
M	Cell wall/membrane/envelope biogenesis	307
N	Cell motility	29
O	Posttranslational modification, protein turnover, chaperones	202
P	Inorganic ion transport and metabolism	390
Q	Secondary metabolites biosynthesis, transport and catabolism	314
R	General function prediction only	0

S	Function unknown	1,186
T	Signal transduction mechanisms	518
U	Intracellular trafficking, secretion, and vesicular transport	80
V	Defense mechanisms	169
W	Extracellular structures	1
X	Mobilome: prophages, transposons	0
Y	Nuclear structure	0
Z	Cytoskeleton	13
	**Total**	**7,018**

*Clusters of Orthologous Groups

Table S7. Hits of the 16S rRNA gene sequence of the strain KPP03845 from EzBioCloud 16S database

**Name**	**Strain**	**Accession**	**Pairwise Similarity (%)**	**Mismatch /Total (nt)**
Streptomyces xanthophaeus	NRRL B-5414	JOFT01000080	100	0/1447
Streptomyces nojiriensis	LMG 20094	AJ781355	100	0/1447
Streptomyces spororaveus	LMG 20313	AJ781370	100	0/1447
Streptomyces lavendulae subsp. *lavendulae*	NRRL B-2774	JOEW01000098	99.9309	1/1447
Streptomyces cirratus	NRRL B-3250	AY999794	99.8618	2/1447
Streptomyces vinaceus	NBRC 13425	AB184394	99.8612	2/1441
Streptomyces sporoverrucosus	NBRC 15458	AB184684	99.8610	2/1439
Streptomyces goshikiensis	NBRC 12868	AB184204	99.8605	2/1434
Streptomyces colombiensis	NRRL B-1990	DQ026646	99.7927	3/1447
Streptomyces virginiae	NRRL ISP-5094	JOAK01000082	99.7234	4/1446
Streptomyces subrutilus	DSM 40445	X80825	99.7234	4/1446
Streptomyces avidinii	NBRC 13429	AB184395	99.7234	4/1446
Streptomyces cinnamonensis	NBRC 15873	AB184707	99.7232	4/1445
Streptomyces manipurensis	MBRL 201	JN560156	99.5156	7/1445
Streptomyces racemochromogenes	NRRL B-5430	DQ026656	98.9627	15/1446
Streptomyces polychromogenes	NBRC 13072	AB184292	98.9583	15/1440
Streptomyces flavotricini	NRRL B-5419	JNXV01000042	98.8935	16/1446
Streptomyces amritsarensis	MTCC 11845	MQUR01000179	98.8935	16/1446
Streptomyces yangpuensis	fd2-tb	LBMK01000002	98.8935	16/1446
Streptomyces yokosukanensis	DSM 40224	KQ948269	98.6178	20/1447
Streptomyces cavourensis	NBRC 13026	AB184264	98.6169	20/1446
Streptomyces lavendulae subsp. *grasserius*	DSM 40385	AY999841	98.5735	20/1402
Streptomyces spiroverticillatus	NBRC 12821	AB249921	98.5487	21/1447
Streptomyces adustus	WH-9	LC026279	98.5487	21/1447
Streptomyces badius	NRRL B-2567	AY999783	98.4796	22/1447
Streptomyces globosus	LMG 19896	AJ781330	98.4786	22/1446
Streptomyces globisporus	NBRC 12867	AB184203	98.4775	22/1445

Streptomyces sindenensis	NBRC 3399	AB184759	98.4775	22/1445
Streptomyces parvus	NBRC 3388	AB184756	98.4775	22/1445
Streptomyces toxytricini	NBRC 12823	AB184173	98.4754	22/1443
Streptomyces pluricolorescens	NBRC 12808	AB184162	98.4743	22/1442
Streptomyces rubiginosohelvolus	NBRC 12912	AB184240	98.4712	22/1439
Streptomyces cremeus	NBRC 12760	AB184124	98.4701	22/1438
Streptomyces lunaelactis	MM109	KM207217	98.4496	22/1419
Streptomyces setonii	NRRL ISP-5322	MUNB01000146	98.4105	23/1447
Streptomyces anulatus	NRRL B-2000	DQ026637	98.4105	23/1447
Streptomyces microflavus	NBRC 13062	AB184284	98.4094	23/1446
Streptomyces katrae	NRRL ISP-5550	JZWV01000648	98.4094	23/1446
Streptomyces puniceus	NBRC 12811	AB184163	98.4094	23/1446
Streptomyces fulvorobeus	NBRC 15897	AB184711	98.4072	23/1444
Streptomyces candidus	NRRL ISP-5141	DQ026663	98.3402	24/1446
Streptomyces flavovirens	NBRC 3716	AB184834	98.2723	25/1447
Streptomyces cyaneofuscatus	NRRL B-2570	JOEM01000050	98.2723	25/1447
Streptomyces griseus subsp. *griseus*	KCTC 9080	M76388	98.2699	25/1445
Streptomyces pratensis	ch24	JQ806215	98.2482	24/1370
Streptomyces araujoniae	ASBV-1	EU792889	98.2407	25/1421
Streptomyces lateritius	LMG 19372	AJ781326	98.2032	26/1447
Streptomyces hypolithicus	HSM10	EU196762	98.2019	26/1446
Streptomyces bacillaris	NBRC 13487	AB184439	98.2019	26/1446

Supplementary Figures

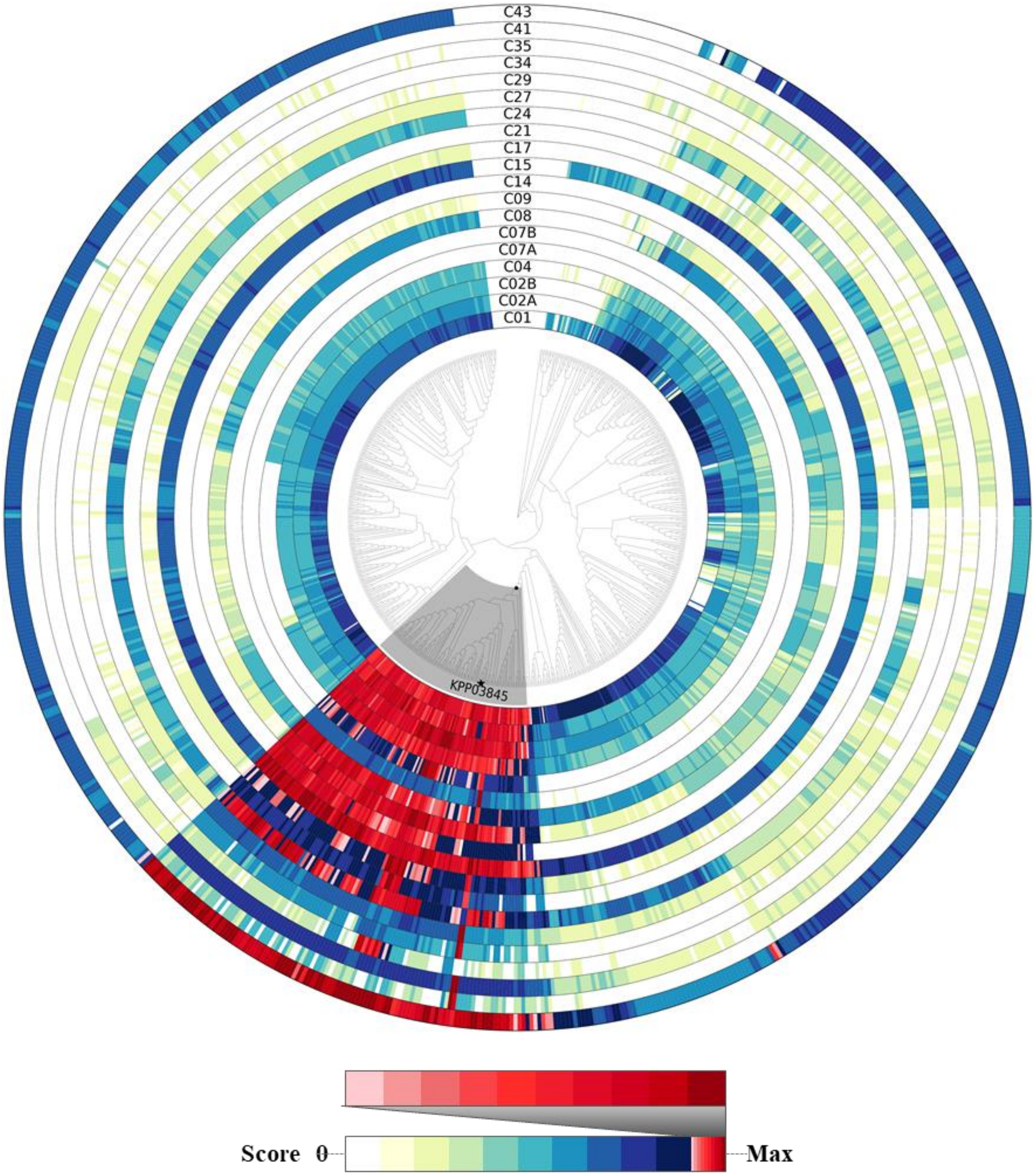

Figure S1. Homology distribution of potential antimicrobial peptides among the highly conserved genes in 762 Actinobacteria genome sequences. Relative ratio to the maximum homology score for each sequence profile was shown in the color gradient on the right. A clade containing the strain KPP03845 and 108 *Streptomyces* spp. was shaded in grey. The strain KPP03845 was indicated with a filled star mark at the terminal node. Significant hits were found in more than 90% of actinobacterial genomes for the sequence profiles of C01, C02A, C02B, C08, C15, C24, and C43. It suggests that these seven sequence profiles, or sequence groups, may be considered as actinobacteria signature proteins.

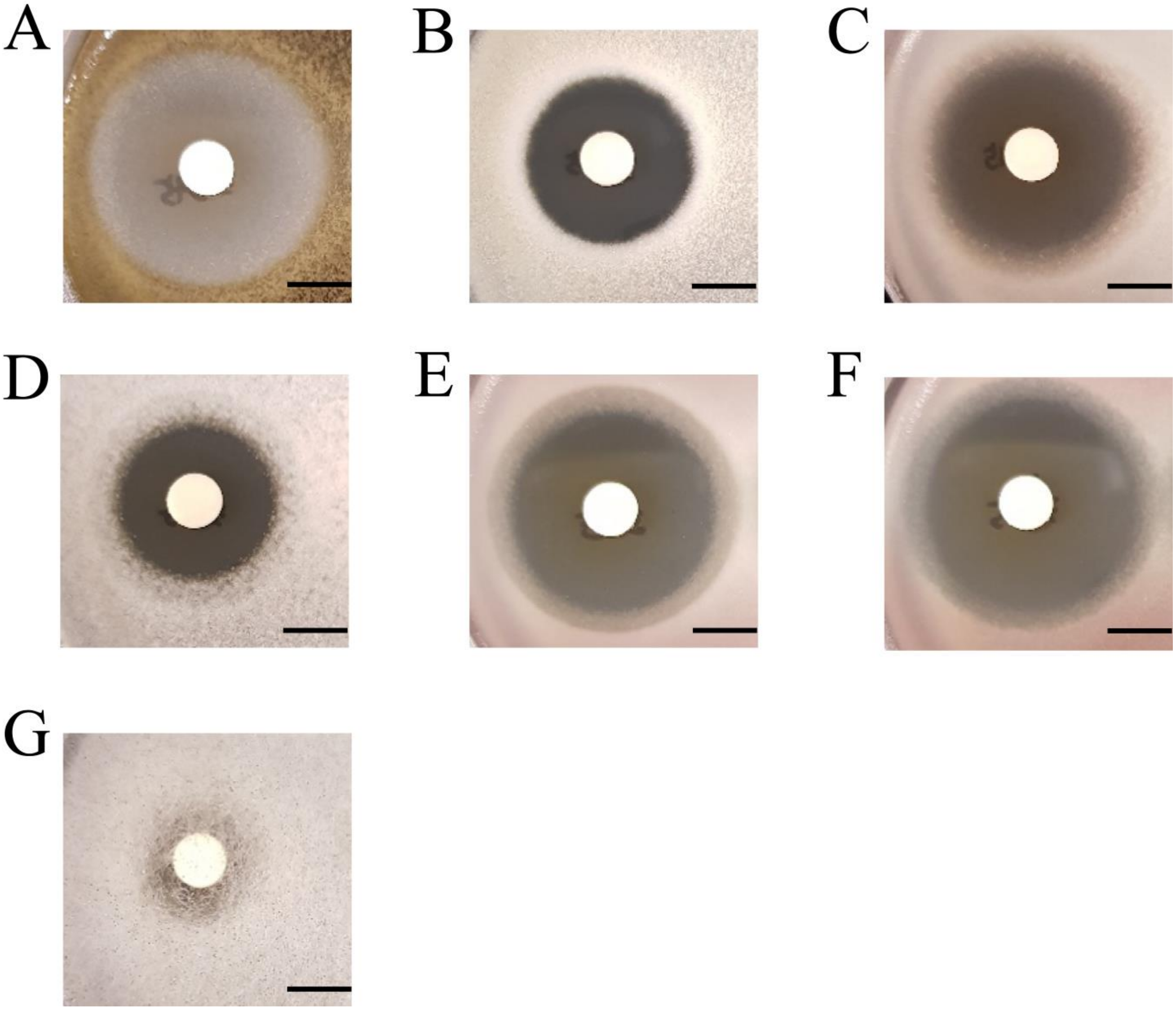

Figure S2. Antifungal activities of the culture filtrate of strain KPP03845 against plant pathogenic fungi. The culture filtrate was concentrated *in vacuo* and re-dissolved in 40 µl of sterile distilled water. The re-dissolved culture filtrate was loaded onto a paper disc. Scale bar represents 10 mm. (A) *Alternaria brassicicola*, (B) *Aspergillus oryzae*, (C) *Colletotrichum gloeosporioides*, (D) *Colletotrichum orbiculare*, (E) *Fusarium oxysporum* f. sp. *cucumerinum*, (F) *Fusarium oxysporum* f. sp. *lycopersici*, (G) *Rhizopus stolonifer* var. *stolonifer*.

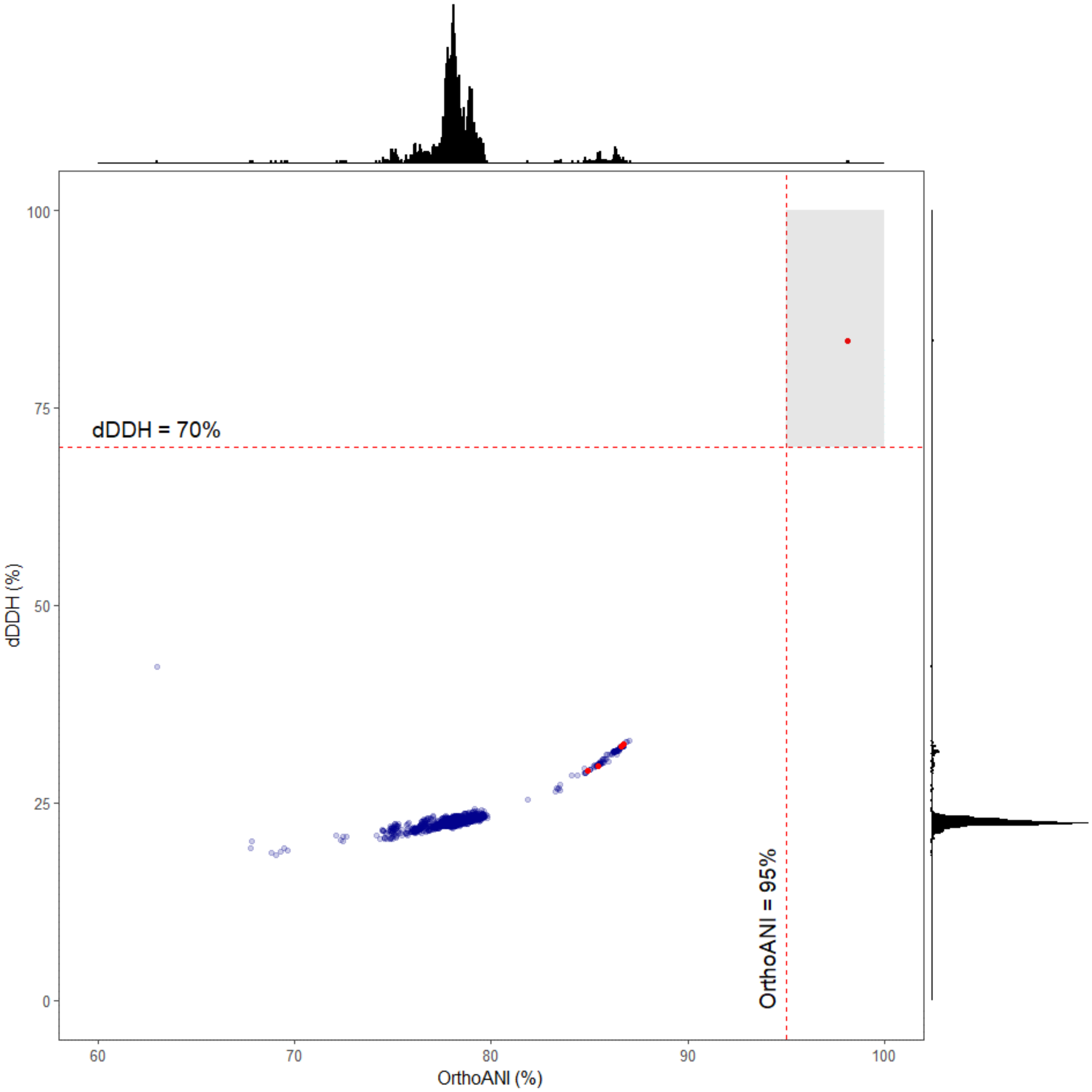

Figure S3. Distribution of genomic relatedness indices between the strain KPP03845 and the 2,061 *Streptomyces* genomes. Pairwise calculations of OrthoANI (X-axis) and dDDH (Y-axis) were shown as a scatter plot. The grey box highlights the 2 strains showing OrthoANI > 95% and dDDH > 70% compared to the strain KPP03845. The top 5 strains showing the highest 16S rDNA sequence similarities were shown as red dots. See Table S2and S7 for more details.

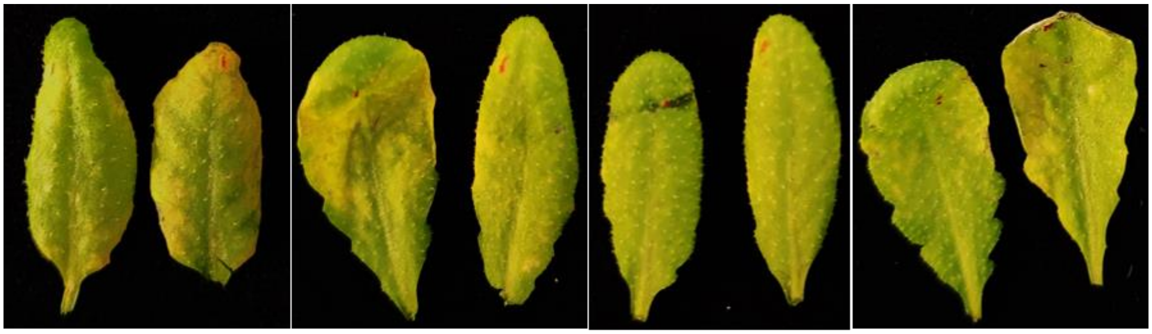

Figure S4. Phenotypic symptoms of *Arabidopsis* leaves caused by *Pst* DC3000 infection after different concentration SHC-AMP.

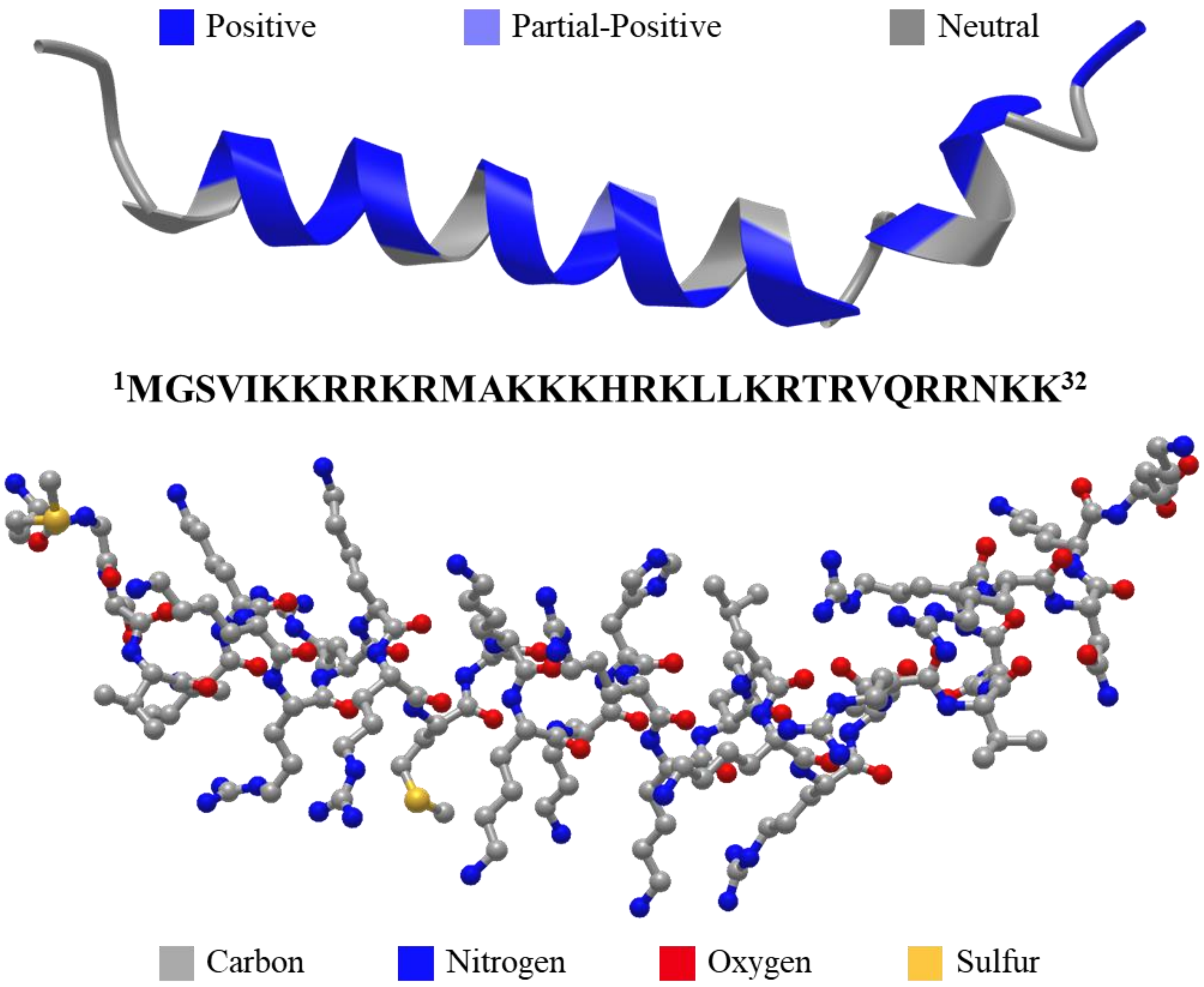

Figure S5. Protein sequence and its homology modeling of SCH-AMP. The tertiary structure of the peptide was graphically represented in the two different styles: ribbon (top) and ball and stick (bottom). For ribbon style representation, basic amino acid residues, arginine (Arg), lysine (Lys), and histidine (His), were displayed in blue colors as shown at the top. For ball and stick representation, four atoms were colored with different colors as indicated at the bottom.

References

Ausmees, N., Wahlstedt, H., Bagchi, S., Elliot, M.A., Buttner, M.J., and Flardh, K. (2007). *SmeA*, a small membrane protein with multiple functions in *Streptomyces* sporulation including targeting of a SpoIIIE/FtsK-like protein to cell division septa. *Mol Microbiol* 65(6)**,** 1458-1473. doi: 10.1111/j.1365-2958.2007.05877.x.

Davis, N.K., and Chater, K.F. (1992). The *Streptomyces coelicolor whiB* gene encodes a small transcription factor-like protein dispensable for growth but essential for sporulation. *Mol Gen Genet* 232(3)**,** 351-358. doi: 10.1007/BF00266237.

Zhang, L., Willemse, J., Claessen, D., and van Wezel, G.P. (2016). *SepG* coordinates sporulation-specific cell division and nucleoid organization in *Streptomyces coelicolor*. *Open Biol* 6(4)**,** 150164. doi: 10.1098/rsob.150164.
